# 2-Methyl­sulfonyl-4-(trifluoro­meth­yl)benzoic acid

**DOI:** 10.1107/S160053681202243X

**Published:** 2012-05-23

**Authors:** Lin-Shan Yao, Bo We, Jin-Sheng Gao

**Affiliations:** a Engineering Research Center of Pesticides of Heilongjiang University, Heilongjiang University, Harbin 150050, People’s Republic of China

## Abstract

In the title mol­ecule, C_9_H_7_F_3_O_4_S, the S and the methyl C atoms of the methyl­sulfonyl group deviate from the benzene ring plane by 0.185 (2) and −1.394 (3) Å, respectively. In the crystal, O—H⋯O hydrogen bonds link the mol­ecules into chains along [201]. Weak C—H⋯O inter­actions further link these chains into layers parallel to the *ac* plane.

## Related literature

For details of the synthesis, see: Cain *et al.* (1998 ▶). 
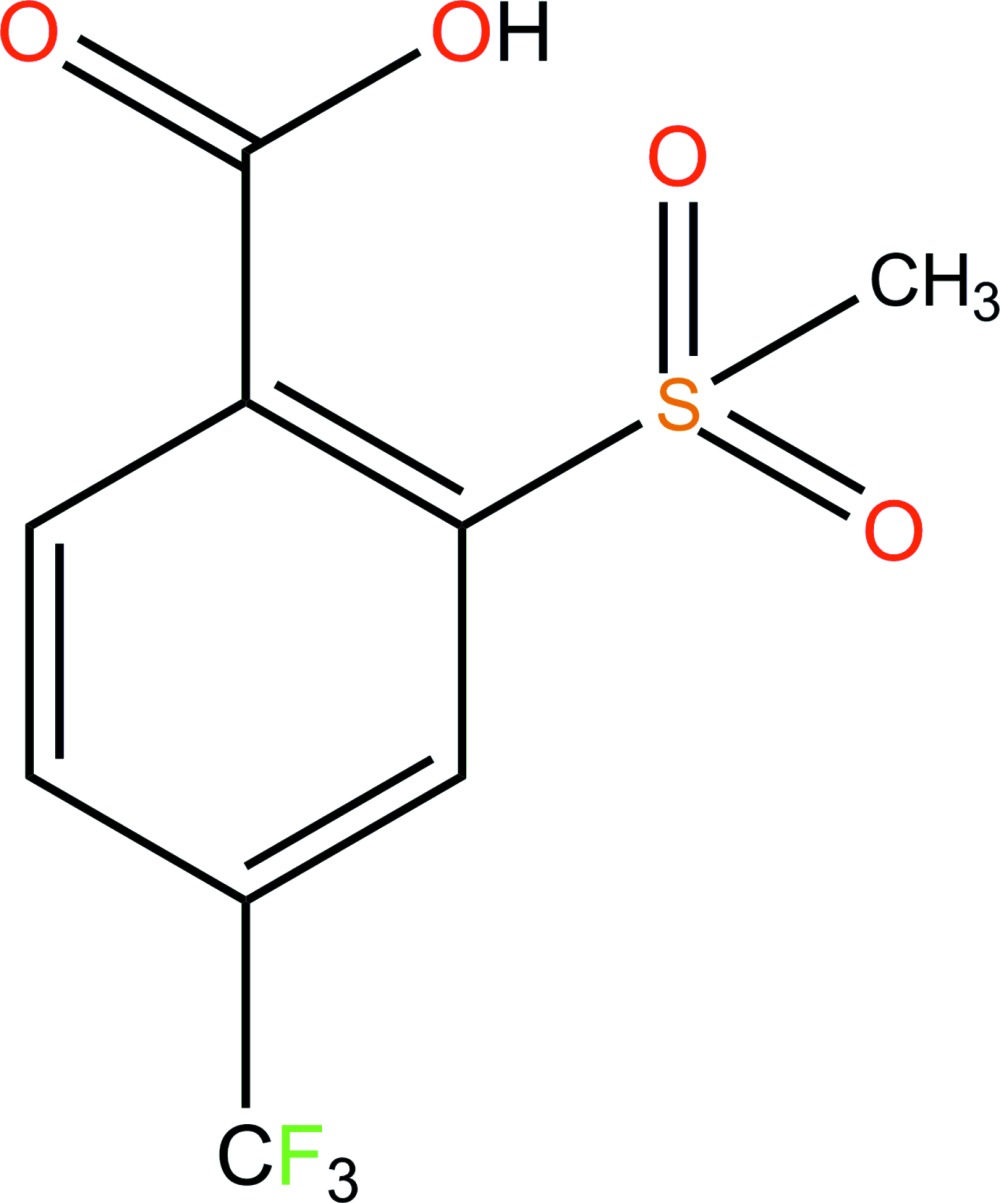


## Experimental

### 

#### Crystal data

C_9_H_7_F_3_O_4_S
*M* *_r_* = 268.21
Monoclinic, 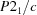

*a* = 5.0804 (10) Å
*b* = 17.345 (4) Å
*c* = 11.576 (2) Å
β = 95.41 (3)°
*V* = 1015.6 (4) Å^3^
*Z* = 4
Mo *K*α radiation
μ = 0.36 mm^−1^
*T* = 293 K
0.39 × 0.32 × 0.22 mm

#### Data collection

Rigaku R-AXIS RAPID diffractometer
Absorption correction: multi-scan (*ABSCOR*; Higashi, 1995 ▶) *T*_min_ = 0.871, *T*_max_ = 0.926
9132 measured reflections
2250 independent reflections
1879 reflections with *I* > 2σ(*I*)
*R*_int_ = 0.029

#### Refinement

*R*[*F* ^2^ > 2σ(*F* ^2^)] = 0.044
*wR*(*F* ^2^) = 0.123
*S* = 1.11
2250 reflections
158 parameters
1 restraint
H atoms treated by a mixture of independent and constrained refinement
Δρ_max_ = 0.39 e Å^−3^
Δρ_min_ = −0.38 e Å^−3^

### 

Data collection: *RAPID-AUTO* (Rigaku, 1998 ▶); cell refinement: *RAPID-AUTO*; data reduction: *CrystalClear* (Rigaku/MSC, 2002 ▶); program(s) used to solve structure: *SHELXS97* (Sheldrick, 2008 ▶); program(s) used to refine structure: *SHELXL97* (Sheldrick, 2008 ▶); molecular graphics: *SHELXTL* (Sheldrick, 2008 ▶); software used to prepare material for publication: *SHELXL97*.

## Supplementary Material

Crystal structure: contains datablock(s) I, global. DOI: 10.1107/S160053681202243X/cv5300sup1.cif

Structure factors: contains datablock(s) I. DOI: 10.1107/S160053681202243X/cv5300Isup2.hkl

Supplementary material file. DOI: 10.1107/S160053681202243X/cv5300Isup3.cml

Additional supplementary materials:  crystallographic information; 3D view; checkCIF report

## Figures and Tables

**Table 1:** Hydrogen-bond geometry (Å, °)

*D*—H⋯*A*	*D*—H	H⋯*A*	*D*⋯*A*	*D*—H⋯*A*
O2—H2⋯O4^i^	0.82 (1)	1.92 (1)	2.725 (3)	169 (4)
C9—H9*B*⋯O3^ii^	0.96	2.35	3.208 (3)	148

Symmetry codes: (i) 
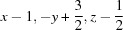
; (ii) 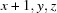.

**Fig. 1.**
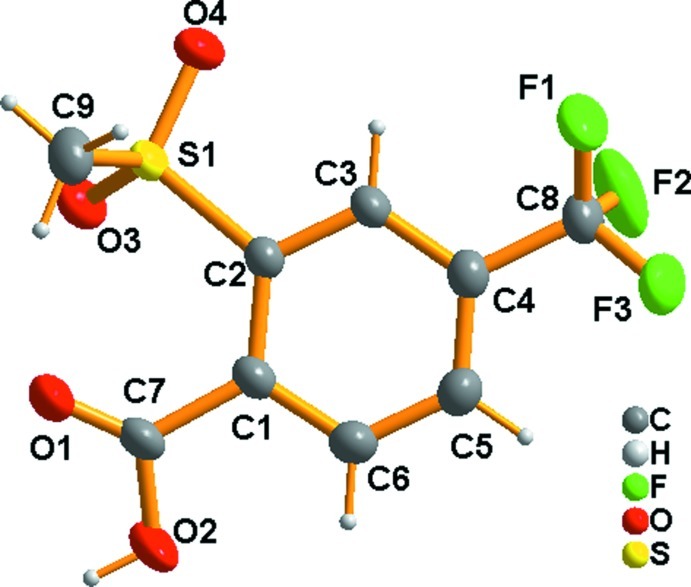
The molecular structure of (I) showing displacement ellipsoids at the 50% probability level for non-H atoms.

**Fig. 2.**
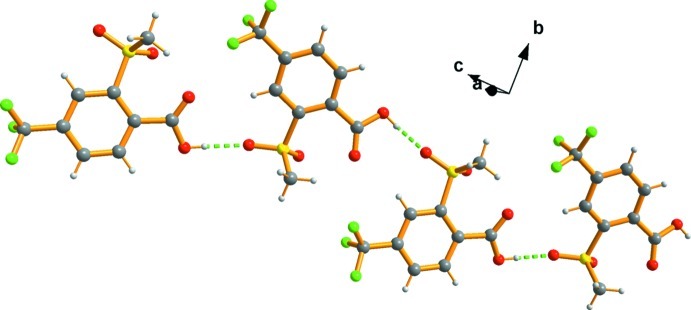
A portion of the crystal packing showing hydrogen-bonded (dashed lines) chains.

## supplementary crystallographic information

### Comment

The title compound, (I), is a intermediate in the synthesis of sulfonylurea herbicides developed and produced by E. I. du Pont de Nemours and Company. Herein, we report its crystal structure.

In (I) (Fig. 1), the S and the methyl C atoms of the methylsulfonyl group deviate from the benzene ring plane at 0.185 (2) and -1.394 (3) Å, respectively. Intermoleculear O—H···O hydrogen bonds (Table 1) link the molecules into chains along [201] (Fig. 2), and weak C—H···O interactions (Table 1) link further these chains into layers parallel to *ac* plane.

### Experimental

The title compound was prepared by the reaction of 2-(methylsulphenyl)-4-trifluoromethylbenzoic acid and hydrogen peroxide in acetic acid at 10 ° (Cain *et al.*, 1998). A colourless crystal suitable for single-crystal X-ray diffraction was obtained by the recrystallization from dichloromethane.

### Refinement

C-bound H atoms were placed in calculated positions and treated as riding on their parent atoms, with C—H = 0.93 – 0.96 Å , and with *U*_iso_(H) = 1.2 – 1.5 *U*_eq_(C). O-bound H atoms were located in a differece Fourier map and were refined with restraint as O—H = 0.82 (1) Å, and *U*_iso_(H) = 1.5*U*_eq_(O).

### Crystal data

**Table:**

C_9_H_7_F_3_O_4_S	*F*(000) = 544
*M**_r_* = 268.21	*D*_x_ = 1.754 Mg m^−^^3^
Monoclinic, *P*2_1_/*c*	Mo *K*α radiation, λ = 0.71073 Å
Hall symbol: -P 2ybc	Cell parameters from 8167 reflections
*a* = 5.0804 (10) Å	θ = 3.0–27.5°
*b* = 17.345 (4) Å	µ = 0.36 mm^−^^1^
*c* = 11.576 (2) Å	*T* = 293 K
β = 95.41 (3)°	Block, colorless
*V* = 1015.6 (4) Å^3^	0.39 × 0.32 × 0.22 mm
*Z* = 4	

### Data collection

**Table:**

Rigaku R-AXIS RAPID diffractometer	2250 independent reflections
Radiation source: fine-focus sealed tube	1879 reflections with *I* > 2σ(*I*)
Graphite monochromator	*R*_int_ = 0.029
ω scan	θ_max_ = 27.5°, θ_min_ = 3.5°
Absorption correction: multi-scan (*ABSCOR*; Higashi, 1995)	*h* = −6→6
*T*_min_ = 0.871, *T*_max_ = 0.926	*k* = −22→22
9132 measured reflections	*l* = −14→14

### Refinement

**Table:**

Refinement on *F*^2^	Primary atom site location: structure-invariant direct methods
Least-squares matrix: full	Secondary atom site location: difference Fourier map
*R*[*F*^2^ > 2σ(*F*^2^)] = 0.044	Hydrogen site location: inferred from neighbouring sites
*wR*(*F*^2^) = 0.123	H atoms treated by a mixture of independent and constrained refinement
*S* = 1.11	*w* = 1/[σ^2^(*F*_o_^2^) + (0.0555*P*)^2^ + 0.7827*P*] where *P* = (*F*_o_^2^ + 2*F*_c_^2^)/3
2250 reflections	(Δ/σ)_max_ = 0.001
158 parameters	Δρ_max_ = 0.39 e Å^−^^3^
1 restraint	Δρ_min_ = −0.38 e Å^−^^3^

### Special details

**Table:**

Geometry. All esds (except the esd in the dihedral angle between two l.s. planes) are estimated using the full covariance matrix. The cell esds are taken into account individually in the estimation of esds in distances, angles and torsion angles; correlations between esds in cell parameters are only used when they are defined by crystal symmetry. An approximate (isotropic) treatment of cell esds is used for estimating esds involving l.s. planes.
Refinement. Refinement of F^2^ against ALL reflections. The weighted R-factor wR and goodness of fit S are based on F^2^, conventional R-factors R are based on F, with F set to zero for negative F^2^. The threshold expression of F^2^ > 2sigma(F^2^) is used only for calculating R-factors(gt) etc. and is not relevant to the choice of reflections for refinement. R-factors based on F^2^ are statistically about twice as large as those based on F, and R- factors based on ALL data will be even larger.

### Fractional atomic coordinates and isotropic or equivalent isotropic displacement parameters (Å^2^)

**Table:**

	*x*	*y*	*z*	*U*_iso_*/*U*_eq_	
C1	0.2822 (5)	0.85011 (13)	0.09229 (19)	0.0319 (5)	
C2	0.4291 (5)	0.80387 (12)	0.17524 (19)	0.0284 (5)	
C3	0.6265 (5)	0.83602 (13)	0.25122 (19)	0.0304 (5)	
H3	0.7242	0.8051	0.3051	0.037*	
C4	0.6780 (5)	0.91494 (13)	0.2467 (2)	0.0337 (5)	
C5	0.5314 (5)	0.96140 (14)	0.1677 (2)	0.0392 (6)	
H5	0.5636	1.0141	0.1658	0.047*	
C6	0.3360 (5)	0.92880 (14)	0.0911 (2)	0.0396 (6)	
H6	0.2386	0.9602	0.0378	0.048*	
C7	0.0778 (5)	0.81875 (14)	0.0022 (2)	0.0353 (5)	
C8	0.8793 (5)	0.94984 (14)	0.3342 (2)	0.0389 (6)	
C9	0.4951 (5)	0.65356 (15)	0.0879 (2)	0.0436 (6)	
H9A	0.4715	0.5992	0.0984	0.065*	
H9B	0.6803	0.6649	0.0889	0.065*	
H9C	0.4062	0.6691	0.0147	0.065*	
F1	1.0869 (4)	0.90478 (10)	0.35927 (19)	0.0655 (5)	
F2	0.7743 (4)	0.96498 (13)	0.43239 (16)	0.0743 (6)	
F3	0.9734 (4)	1.01622 (10)	0.29917 (18)	0.0648 (5)	
O1	0.0598 (4)	0.75293 (11)	−0.02770 (17)	0.0511 (5)	
O2	−0.0822 (4)	0.87346 (13)	−0.0433 (2)	0.0594 (6)	
H2	−0.187 (6)	0.853 (2)	−0.092 (3)	0.089*	
O3	0.0826 (3)	0.69334 (11)	0.19817 (16)	0.0409 (4)	
O4	0.5177 (4)	0.68310 (10)	0.30716 (16)	0.0420 (4)	
S1	0.36264 (11)	0.70384 (3)	0.20026 (5)	0.02965 (18)	

### Atomic displacement parameters (Å^2^)

**Table:**

	*U*^11^	*U*^22^	*U*^33^	*U*^12^	*U*^13^	*U*^23^
C1	0.0313 (12)	0.0354 (12)	0.0275 (10)	−0.0014 (9)	−0.0052 (9)	−0.0003 (9)
C2	0.0292 (11)	0.0281 (10)	0.0270 (10)	−0.0019 (8)	−0.0027 (9)	−0.0010 (8)
C3	0.0292 (11)	0.0307 (11)	0.0298 (10)	−0.0012 (9)	−0.0056 (9)	−0.0011 (9)
C4	0.0343 (13)	0.0323 (11)	0.0340 (11)	−0.0041 (9)	0.0002 (10)	−0.0039 (9)
C5	0.0465 (15)	0.0295 (11)	0.0402 (13)	−0.0049 (10)	−0.0025 (11)	0.0017 (9)
C6	0.0461 (15)	0.0348 (12)	0.0358 (12)	−0.0008 (10)	−0.0079 (11)	0.0054 (10)
C7	0.0363 (13)	0.0409 (13)	0.0264 (11)	−0.0014 (10)	−0.0080 (10)	0.0021 (9)
C8	0.0381 (14)	0.0344 (12)	0.0426 (13)	−0.0063 (10)	−0.0053 (11)	−0.0041 (10)
C9	0.0411 (14)	0.0387 (13)	0.0500 (15)	0.0002 (11)	−0.0014 (12)	−0.0120 (11)
F1	0.0520 (11)	0.0479 (10)	0.0892 (14)	0.0019 (8)	−0.0329 (10)	−0.0072 (9)
F2	0.0700 (14)	0.1067 (17)	0.0459 (10)	−0.0298 (12)	0.0039 (9)	−0.0328 (10)
F3	0.0673 (12)	0.0446 (9)	0.0773 (12)	−0.0257 (8)	−0.0208 (10)	0.0066 (9)
O1	0.0628 (14)	0.0392 (10)	0.0453 (10)	−0.0044 (9)	−0.0255 (10)	−0.0005 (8)
O2	0.0596 (14)	0.0481 (12)	0.0622 (13)	0.0101 (10)	−0.0377 (11)	−0.0121 (10)
O3	0.0279 (9)	0.0490 (10)	0.0441 (10)	−0.0075 (7)	−0.0047 (8)	0.0036 (8)
O4	0.0404 (10)	0.0411 (10)	0.0410 (10)	−0.0038 (7)	−0.0135 (8)	0.0091 (7)
S1	0.0264 (3)	0.0294 (3)	0.0313 (3)	−0.0036 (2)	−0.0070 (2)	0.0017 (2)

### Geometric parameters (Å, º)

**Table:**

C1—C6	1.392 (3)	C7—O1	1.194 (3)
C1—C2	1.410 (3)	C7—O2	1.326 (3)
C1—C7	1.502 (3)	C8—F1	1.322 (3)
C2—C3	1.387 (3)	C8—F3	1.325 (3)
C2—S1	1.796 (2)	C8—F2	1.326 (3)
C3—C4	1.396 (3)	C9—S1	1.753 (3)
C3—H3	0.9300	C9—H9A	0.9600
C4—C5	1.383 (3)	C9—H9B	0.9600
C4—C8	1.498 (3)	C9—H9C	0.9600
C5—C6	1.387 (4)	O2—H2	0.816 (10)
C5—H5	0.9300	O3—S1	1.4325 (18)
C6—H6	0.9300	O4—S1	1.4484 (18)
			
C6—C1—C2	118.2 (2)	O2—C7—C1	112.0 (2)
C6—C1—C7	118.1 (2)	F1—C8—F3	106.1 (2)
C2—C1—C7	123.6 (2)	F1—C8—F2	107.8 (2)
C3—C2—C1	120.5 (2)	F3—C8—F2	106.0 (2)
C3—C2—S1	114.94 (17)	F1—C8—C4	112.9 (2)
C1—C2—S1	124.32 (17)	F3—C8—C4	112.8 (2)
C2—C3—C4	119.8 (2)	F2—C8—C4	110.8 (2)
C2—C3—H3	120.1	S1—C9—H9A	109.5
C4—C3—H3	120.1	S1—C9—H9B	109.5
C5—C4—C3	120.3 (2)	H9A—C9—H9B	109.5
C5—C4—C8	120.2 (2)	S1—C9—H9C	109.5
C3—C4—C8	119.3 (2)	H9A—C9—H9C	109.5
C4—C5—C6	119.6 (2)	H9B—C9—H9C	109.5
C4—C5—H5	120.2	C7—O2—H2	107 (3)
C6—C5—H5	120.2	O3—S1—O4	116.25 (11)
C5—C6—C1	121.5 (2)	O3—S1—C9	112.01 (13)
C5—C6—H6	119.3	O4—S1—C9	107.14 (13)
C1—C6—H6	119.3	O3—S1—C2	108.76 (11)
O1—C7—O2	122.8 (2)	O4—S1—C2	106.40 (11)
O1—C7—C1	125.2 (2)	C9—S1—C2	105.64 (12)

### Hydrogen-bond geometry (Å, º)

**Table:**

*D*—H···*A*	*D*—H	H···*A*	*D*···*A*	*D*—H···*A*
O2—H2···O4^i^	0.82 (1)	1.92 (1)	2.725 (3)	169 (4)
C9—H9*B*···O3^ii^	0.96	2.35	3.208 (3)	148

Symmetry codes: (i) *x*−1, −*y*+3/2, *z*−1/2; (ii) *x*+1, *y*, *z*.
